# Debate on the compositions of influenza B in northern hemisphere seasonal influenza vaccines

**DOI:** 10.1186/s13756-019-0631-2

**Published:** 2019-10-28

**Authors:** Guozhong He, Pengfei Yang, Qingli Yan, Chenglong Xiong

**Affiliations:** 1 Institute of Health, Kunming Medical University, Kunming, 650031 China; 2 Huai’an Center for Disease Control and Prevention, Huai’an, 223005 China; 3 Department of Public Health Microbiology, School of Public Health, Fudan University, Shanghai, 200032 China; 4 School of Public Health, Fudan University, Key Laboratory of Public Health Safety, Ministry of Education, Shanghai, 200032 China

**Keywords:** Seasonal influenza, Northern hemisphere, Vaccine, Influenza B

## Abstract

**Background:**

Annual influenza vaccination is the most effective way to prevent influenza. Influenza vaccines have traditionally included the hemagglutinins (HA) and neuraminidases (NA) from the two A viruses (H1N1 and H3N2) and either B Yamagata or B Victoria. Mismatches between circulating isolates of influenza B and the vaccines are very common. Taking 2017/2018 winter in northern hemisphere as an example, this study was designed to find out the reasons for mismatch between the trivalent influenza vaccine (TIV) and most of the epidemic isolates at that time, and to discuss if there are some optimized programs for seasonal influenza vaccines.

**Methods:**

HA and NA sequences of the seasonal isolates circulating from December 1, 2017 to February 28, 2018, and in the previously other 7 winters in northern hemisphere from Global Initiative on Sharing All Influenza Data (GISAID) and the influenza database of National Center for Biotechnology Information (NCBI). Phylogenetic trees and genetic distances were constructed or calculated by using MAFFT and MEGA 6.0 software.

**Results:**

Influenza B composition in the TIV recommendation mismatched most of circulating viruses in 2017/2018 winter; the vaccine strain was from the B/Victoria lineage, while most of epidemic isolates were from the B/Yamagata lineage. The epidemic lineage of influenza B reached its peak a little late in the previous winter might be responsible for this mismatch. During 2010–2018, the mean genetic distances between epidemic isolates of influenza A (H1N1 and H3N2) and the vaccines were no higher than 0.02375 ± 0.00341 in both HA and NA. However, concerning influenza B virus, when forecasting done well, the mean genetic distances between epidemic isolates and the vaccines were no higher than 0.02368 ± 0.00272; otherwise, the distances could reach 0.13695 ± 0.00238.

**Conclusion:**

When applying quadrivalent influenza vaccines (QIVs) for vaccination, the recommendations of compositions for influenza B could be altered and assessed once in 3 or 4 years; when economic burden was considered intensively and TIVs were utilized, the recommended compositions for influenza B could be announced in April or May, rather than in February or March as now.

## Background

Influenza (flu) is a contagious acute respiratory infection, causing considerable global morbidity, mortality and economic burden every year. According to a new estimate based on a robust, multinational survey, between 291,000 and 646,000 people worldwide die from seasonal influenza-related respiratory illnesses annually, higher than the previous estimate of 250,000 to 500,000 [1, 2]. Influenza viruses, the pathogens which are responsible for these infections, belong to the Orthomyxoviridae family and are widely distributed among mammals and birds. They are divided into 4 different types A, B, C, and D based on variation in their expressed matrix and nucleoproteins, and the vast majority of human disease is caused by types A and B [3, 4]. Since 1977, four sub-types or lineages viruses–two A viruses (H1N1 and H3N2) and two B viruses (B Yamagata and B Victoria) co-circulate annually and globally [5].

It is widely accepted that an annual influenza vaccination is the most effective way to prevent influenza [6]. Influenza vaccines have traditionally included the hemagglutinins and neuraminidases from the two influenza A viruses (H1N1 and H3N2) and either Yamagata lineage or Victoria lineage of influenza B virus. In both influenza A and B, there exists possibilities of mismatch between epidemic isolates and vaccine strains. When antigens in a vaccine match those of circulating isolates, the vaccine is considered as effective. However, antigens contained in the vaccine do not always match the circulating ones. Mismatched seasons may lead to reduced uptake of influenza vaccination and severe influenza epidemics, and estimating the prevention that can be achieved during mismatched influenza seasons is of prime public health importance [7–9]. In this study, based on the genetic features of the circulating viruses in northern hemisphere in the winter of 2017/2018, we aimed to discuss if there are some optimized programs for seasonal influenza vaccines.

## Methods

### Study design and nucleotide sequences collection

In northern hemisphere, human transmission of seasonal influenza occurs in winter months but the exact time and duration usually varies by country and by year. In the USA, China, and many European countries, winter epidemic can begin as early as October, but it does not reach the peak until a rapid increase occurs in December. Typically, the peaks can last until February of the next year, and then the winter in northern hemisphere is often determined from December to the next February [10]. Therefore, in this study, we collected HA and NA sequences of the seasonal isolates from December 1, 2017 to February 28, 2018 in both Global Initiative on Sharing All Influenza Data (GISAID, http://platform.gisaid.org/epi3/frontend#3b9e09) and the influenza database of National Center for Biotechnology Information (NCBI, https://www.ncbi.nlm.nih.gov/genomes/FLU/Database/nph-select.cgi?go=database). Information about the vaccine composition was referred to the Vaccine Position Papers in website of WHO (http://www.who.int/immunization/documents/positionpapers/en/). Recommendation compositions of the trivalent influenza vaccine (TIV) for 2017/2018 winter included an A/Michigan/45/2015pdm09-like virus (H1N1), an A/Hong Kong/4801/2014-like virus (H3N2) and a B/Brisbane/60/2008-like (from the B/Victoria lineage) virus.

Recommendations for influenza vaccine composition are updated annually [5, 11]. The Global Influenza Surveillance and Response System (GISRS) of World Health Organization (WHO) is responsible for rolling the identification of circulating influenza viruses. Detection for HA and NA genes of influenza viruses by reverse transcription polymerase chain reaction, and an additional procedure includes consideration of antigenic mismatch between vaccine strains and actual epidemic strains, enables people to early identify the strain divergences from the recommended vaccine this year and to forecast the new composition for next winter [5, 12, 13]. The whole procedure will be completed in February or March, and the recommended compositions of vaccine for the next winter in northern hemisphere are announced soon [11]. Hence, exactly identifying the genetic and antigenic characteristics of the isolates established in the former winter is essential to accurately forecast the compositions of vaccine for the next year. In order to find out the reasons for match or mismatch between vaccines and epidemic strains, we tracked the epidemic isolates in the northern hemisphere, mainly including Asia, North America, and Europe from December 2016 to February 2018.

Seasonal epidemics associated viruses, including the isolates of influenza A(H1N1), A(H3N2), and influenza B viruses and the corresponding ones in the recommendation compositions of TIVs in the previous 8 years were also analyzed, and the isolation sites were restricted to the Northern Hemisphere. According to their lineage/subtype, as well as the year, HA and NA sequences of the seasonal epidemic isolates in northern hemisphere were downloaded from GISAID and the influenza database of NCBI. After clicking Species/Abbrv button in Molecular Evolutionary Genetic Analysis (MEGA) software version 6.0, the first 500 sequences of each lineage/subtype and each year are remained for the purpose of random sampling, and the sequences are all remained when less than 500.

### Phylogenetic analysis

Sequences were aligned using FFT-NS-2 methods of program MAFFT. Phylogenetic trees were constructed using Neighbor-joining method based on the Kimura 2-parameter model as implemented in MEGA 6.0, with the settings as gamma distributed and complete deletion for missing data treatment. Robustness of the trees was assessed using bootstrap analysis of 700 replicates.

Mean genetic distances between a single/group of vaccine(s) and the groups of epidemic isolates in different years or periods in the Northern Hemisphere were calculated by MEGA 6.0 under the set of Kimura 2-parameter model, gamma distributed, 700 bootstrap replications, and complete deletion for missing data treatment.

## Results

### Match or mismatch between vaccine and epidemic isolates

**Fig. 1:**
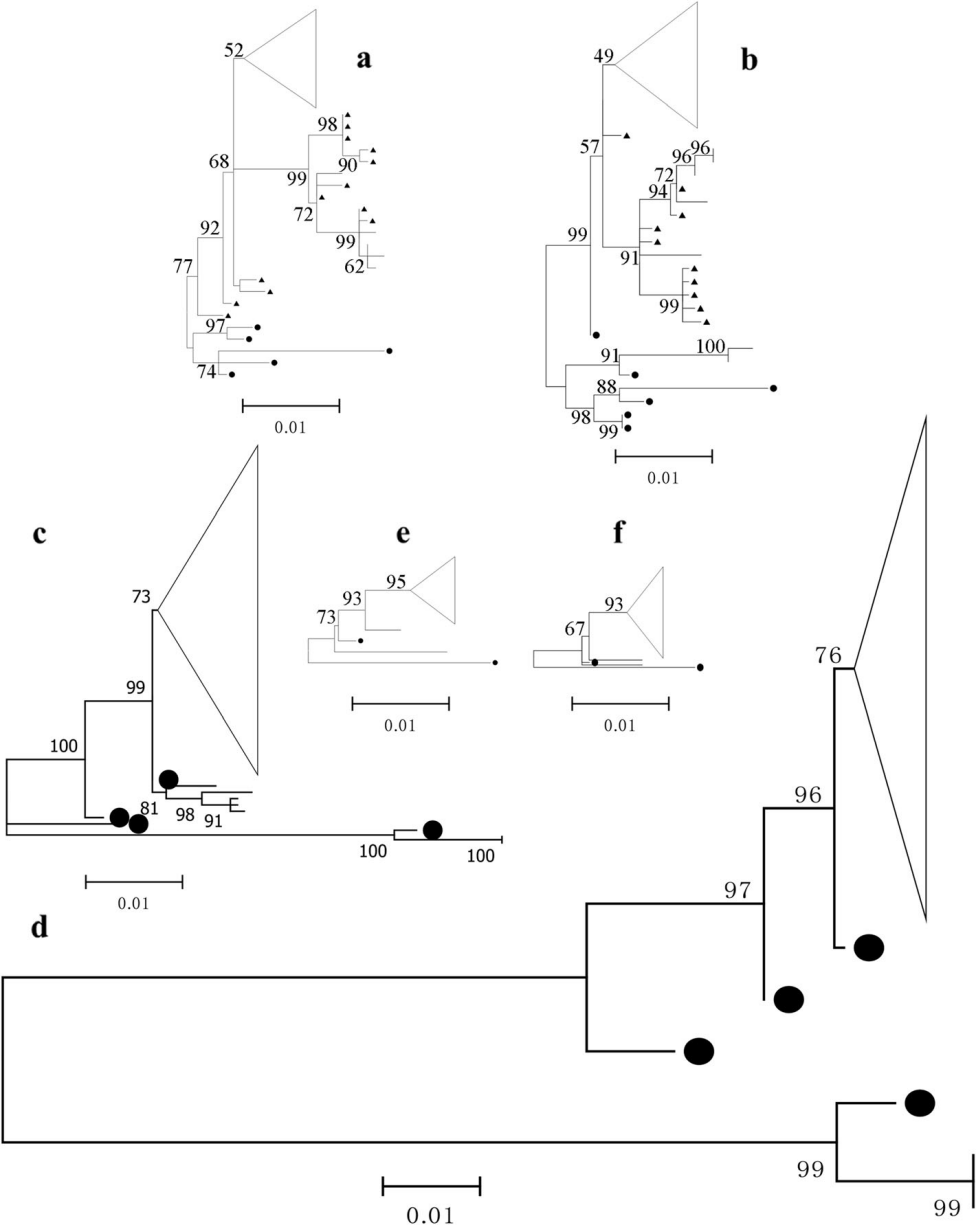
Phylogenetic trees of HA and NA of the seasonal influenza viruses in 2017/2018 winter on the same evolutionary scale. **a**- **f** correspond to N2, H3, NA of influenza B virus, HA of influenza B virus, H1, N1, respectively. Branches labelled by black dots are sequences of recommended compositions in the 8 years (2010/2011–2017/2018) vaccines

Phylogenetic trees demonstrated an interesting image when dealt on the same evolutionary scale; the branches of two trees of influenza B virus were as almost 5 times width as those of other trees. The epidemic isolates of influenza B virus demonstrated quite a difference from the corresponding compositions in the TIV than those of others (Fig. 1). Data of genetic distances also proved these results (Tables 1 and 2). Influenza B virus in the TIV recommendation mismatched most circulating viruses in 2017/2018 winter; the vaccine strain was from the B/Victoria lineage, while most of epidemic isolates were from the B/Yamagata lineage.

**Table 1:** Records of epidemic isolate in 8 winters and the compositions of influenza B in northern hemisphere seasonal influenza vaccines, 2010–2018

Year	HA				NA				Vaccine strain	Lineage
	Total	VC^a^	YM^b^	Unk^c^	Total	VC	YM	Unk		
10/11	794	467	114	213	704	436	102	166	B/Brisbane/60/2008-like	VC
11/12	688	197	204	287	567	176	168	223	B/Brisbane/60/2008-like	VC
12/13	1208	234	709	265	699	171	458	70	B/Wisconsin/1/2010-like	YM
13/14*	584	115	245	224	503	112	219	172	B/Massachusetts/2/2012-like	YM
14/15*	1196	109	898	189	849	107	651	91	B/Massachusetts/2/2012-like	YM
15/16*	2427	1269	739	419	1948	1098	714	136	B/Phuket/3073/2013-like	YM
16/17*	1624	822	652	150	1406	746	599	61	B/Brisbane/60/2008-like	VC
17/18*	5575	722	4300	553	4269	623	3483	163	B/Brisbane/60/2008-like	VC

^**a**^VC, influenza B/Victoria lineage viruses, ^**b**^ YM, influenza B/Yamagata viruses,

^**c**^Unk, unknown lineage influenza B viruses

* Only the recommended compositions of influenza B in trivalent influenza vaccines (TIVs) for northern hemisphere seasonal flu were listed

Data updated on September 10, 2019

**Table 2:** Genetic distances between circulating viruses and vaccines in northern hemisphere, 2010–2018

	Vaccines vs Isolates	HA	NA
H3N2	17/18 vs 17/18 (*n* = 500)	0.01229 ± 0.00239	0.01782 ± 0.00354
	10–18 (n = 5) vs 17/18 (*n* = 500)	0.01940 ± 0.00267	0.01733 ± 0.00226
	10–18 (n = 5) vs 13–18 (*n* = 4000)	0.01677 ± 0.00302	0.01323 ± 0.00212
H1N1	17/18 vs 17/18 (n = 500)	0.01516 ± 0.00299	0.00858 ± 0.00224
	10–18 (*n* = 2) vs 17/18 (n = 500)	0.02375 ± 0.00341	0.01839 ± 0.00309
	10–18 (n = 2) vs 10–18 (n = 4000)	0.01434 ± 0.00279	0.01495 ± 0.00326
Flu B	^*^V17/18 vs ^#^Y17/18 (n = 500)	0.11356 ± 0.00261	0.06874 ± 0.00470
	V10–18 (*n* = 1) vs V17/18 (n = 500)	0.01367 ± 0.00272	0.01331 ± 0.00197
	V10–18 (n = 1) vs V10–18 (*n* = 2122, 2022)	0.01167 ± 0.00218	0.00958 ± 0.00137
	Y10–18 (*n* = 3) vs Y17/18 (n = 500)	0.02261 ± 0.00226	0.02024 ± 0.00260
	Y10–18 (n = 3) vs Y10–18 (*n* = 2563, 2489)	0.01638 ± 0.00236	0.01769 ± 0.00198
	Y15/16 vs V15/16 (n = 500)	0.13695 ± 0.00238	0.07425 ± 0.00328
	Y10–15 (n = 2) vs Y17/18 (n = 500)	0.02368 ± 0.00272	0.02261 ± 0.00315
	Y10–15 (n = 2) vs Y10–18 (*n* = 2563, 2489)	0.02362 ± 0.00218	0.02158 ± 0.00129
	Y15/16 vs Y17/18 (n = 500)	0.01224 ± 0.00243	0.00926 ± 0.00218
	Y15/16 vs Y10–18 (n = 2563, 2489)	0.00721 ± 0.00144	0.00715 ± 0.00101

^*^ V, influenza B/Victoria lineage viruses, ^**#**^ Y, influenza B/Yamagata viruses

It showed the great divergences between the vaccine strains and the seasonal isolates in 2017/2018 and 2015/2016 when the compositions of flu B in the recommended TIVs mismatched the epidemic isolates in the Northern Hemisphere

Throughout the winter, isolates of influenza A(H1N1) were genetically similar to the selected vaccine virus A/Michigan/45/2015pdm09-like, with the mean genetic distances between them being 0.01516 ± 0.00299(HA) and 0.00858 ± 0.00224(NA). Mean genetic distances between the seasonal isolates of influenza A(H3N2) and the selected vaccine virus A/Hong Kong/4801/2014-like were 0.01229 ± 0.00239(HA) and 0.01782 ± 0.00354(NA) (Table 2).

### Reasons for mismatch between the recommended flu B compositions of vaccine and the epidemic isolates

**Fig. 2:**
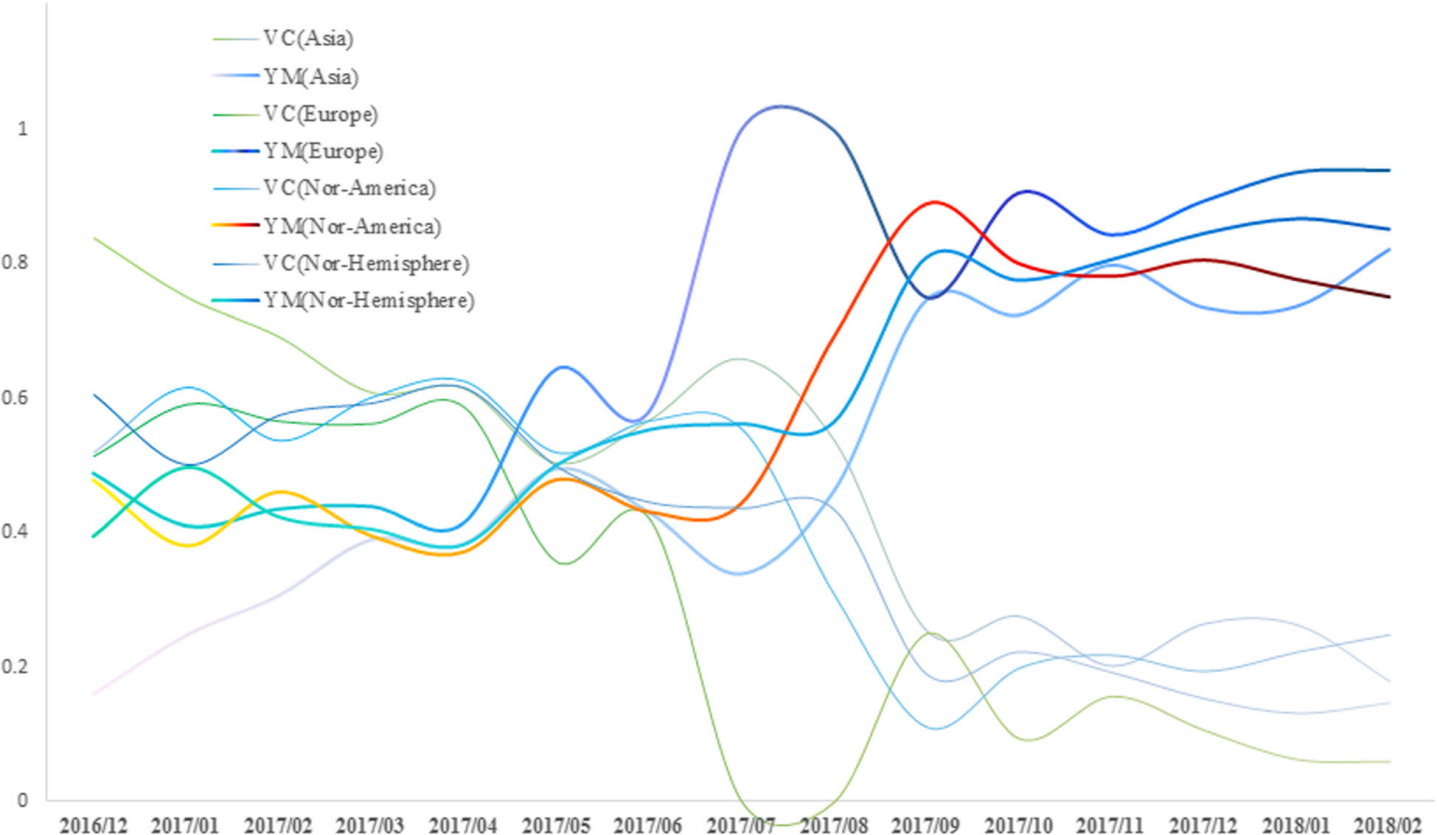
Proportions of influenza B/Victoria and Yamagata lineages in northern hemisphere from December 2016 to February 2018. VC, influenza B/Victoria lineage; YM, influenza B/Yamagata lineage. Curves in this figure reflected the rising (YM) and falling (VC) trends from December 2016 to February 2018 in northern hemisphere. In addition, the inflexions of these curves generally occurred between April and May

As manifested in Fig. 2, the proportion of B/Victoria lineage continued to rise from the winter epidemic of 2016/2017 in most continents of northern hemisphere other than Asia, according to this trend, B/Victoria lineage was strong likely to be the predominate in next winter. It is understandable that B/Brisbane/60/2008-like, which is a strain from B/Victoria lineage, would be recommended as the influenza B composition in the TIV. Unexpectedly, after April, the proportion of B/Victoria lineage undergone a significantly continuous decline, whereas isolates from B/Yamagata lineage had been climbing steadily; the alternation performed drastically in July and August, and then the Yamagata lineage of flu B became the dominated one in the winter epidemic of 2017/2018.

### Seasonal epidemics associated viruses in the previous 8 years in northern hemisphere

According to the calculation, as far as influenza A(H1N1) and A(H3N2) were concerned, the mean genetic distances between epidemic isolates and the vaccines were no higher than 0.02375 ± 0.00341 in both HA and NA. With regards to influenza B virus, when forecasting done well, somewhat like to bet rightly, the mean genetic distances between epidemic isolates and the vaccines were no higher than 0.02368 ± 0.00272, and they might protect the vaccinator well against the epidemic isolates. Otherwise, when forecasting did not work well, the distances between the vaccine strains and the circulating isolates could reach 0.13695 ± 0.00238, as that of the winter epidemic in 2015/2016. Genetic distances also demonstrated that the B/Phuket/3073/2013-like virus, which was recommended as the composition of flu B vaccine for 2015/2016 seasonal epidemic, might be the right one for the flu B in 2017/2018 winter (Table 2).

Moreover, viruses of B/Wisconsin/1/2010-like for winter of 2012/2013, B/Massachusetts/02/2012-like for winters of 2013/2014 and 2014/2015, and B/Phuket/3073/2013-like for winter of 2015/2016, were all of the B/Yamagata lineage. The mean genetic distances between them and the similar lineage seasonal isolates established from the recent eight years were 0.01638 ± 0.00236(HA) and 0.01769 ± 0.00198(NA). The mean genetic distances between B/Brisbane/60/2008-like virus, which was recommended for winters of 2010–2012, 2016/2017 and 2017/2018, and the similar lineage seasonal isolates established from the recent eight years were 0.01167 ± 0.00218(HA) and 0.00958 ± 0.00137(NA) (Table 2). It indicated that isolates of influenza B virus were very heterogeneous between B/Yamagata and B/Victoria lineage, but they shared close phylogenetic relationships within the same lineage.

## Discussion

Although varying from year-to-year, influenza B generally causes up to one-third of influenza infections each season. Some studies focused on specific areas have also shown that it may be the predominant type every four years approximately [5, 14, 15]. In early outbreak of the 2017/2018 winter, seasonal flu in the Northern Hemisphere was caused mainly by influenza B viruses; according to this study and other reports [16–18], epidemic isolates of them mismatched the recommendation compositions of vaccine in most northern hemisphere areas. It implies that the recommended TIV for 2017/2018 winter possibly could not protect the vaccinated well against the epidemic influenza B isolates. This was exactly the case in both the mainland of China and the Hong Kong Special Administrative Region (SAR), where the recommendation of TIV was applied. Until mid-January of 2018, more than 80% of seasonal infections reported in both areas were of influenza B [16, 17].

Two antigenically distinct influenza B virus lineages had been reported to co-circulate since 2001, from then on, selecting the B strain for inclusion in TIVs has variable success, and the vaccine strains that did not match the circulating ones have been reported in seven seasonal epidemics in the Northern Hemisphere [8, 10, 14, 19]. A quadrivalent influenza vaccine (QIV), which included both lineages of influenza B virus, might be the most suitable for prevention seasonal flu in the Northern Hemisphere, even if there is a less accurate prediction about them. As we analyzed, isolates within the same lineage often share very close phylogenetic relationships. This happens partly because within a single host species (i.e., human), influenza B virus need not to be subjected to multiple immune pressures from various species of hosts as that undergone by influenza A virus when the host shift occurs [20]. Partly because there are no reassortment mechanism for HA or NA segment of influenza B viruses as that for influenza A viruses so far [7, 21, 22]. Only by accumulating immune pressure from a single species of host, acquisition of variation can be extremely limited. This meant, unless the forecasting was failure at the lineage level, vaccines for influenza B might cross-react well with the epidemic isolates. This feature is beneficial to application of QIVs, because it does not need to be very strict in selecting the composition of influenza B virus for recommendations, as long as the two lineages are included simultaneously.

Based on the above analysis, we here approve of the approach that to include both lineages of influenza B strains in QIVs, as many researchers suggested [19, 23–26]. For example, B/Brisbane/60/2008(B/Victoria lineage) and B/Phuket/3073/2013(B/Yamagata lineage) might be conceived as two compositions of influenza B vaccines over a considerable period. The compositions do not need to be altered annually but once in 3 or 4 years. This may greatly reduce the time cost for forecasting compositions of flu B vaccines. Indeed, since 2010, B/Brisbane/60/2008-like virus had been recommended for 4 winters of 2010/2011, 2011/2012, 2016/2017, and 2017/2018, and another virus of B/Massachusetts/02/2012-like had been recommended twice for seasonal epidemics of 2013/2014 and 2014/2015 [27].

However, QIVs are more expensive than TIVs, and there must be an increased costs associated with the use of them [24, 25]. Especially in those underdeveloped countries, the costs may be formidable to most of the population. In most continents of northern hemisphere, an epidemic lineage of influenza B often reaches its peak a little late at the end of the usual winter outbreak [10]. This brings many uncertainties for forecasting the influenza B composition of a TIV in advance for the next winter. Acquisition of immunity against temporary circulating influenza B viruses might be responsible for these uncertainties of match or mismatch [7, 19, 23]. Both TIVs vaccination and natural infection can lead to an acquisition of immunity. Usually, in a seasonal outbreak, as time goes on, vaccination population and natural infection population will simultaneously increase, and the immunized population against the circulating viruses will increase subsequently. A higher baseline of immunized population will inevitably produce a relative larger immune pressure to the temporary viruses; this may result in a pathogenic alternative of influenza B virus, for reasons of there are only two lineages of them so far [28–30].

According to the compositional fluctuation of the circulating influenza B virus annually, we here urge an alternative approach that GISRS could recommended the compositions of the TIVs later in April or May, rather than in February or March. In this way, the possibility of accurately forecasting the predominant viruses for the next season can be greatly improved, and mismatches could possibly be avoided. Nowadays, technology for vaccine producing is becoming more and more reliable [31–33]. There should not be technical obstacles in producing a large amount of vaccines in a relatively short time (2–3 months later). Through the technology of virus rescue to introduce the six internal genes from a donor strain with clear genetic background, low-temperature-adaptable influenza vaccines can be produced. Not only can it be rapidly propagated under a relatively low temperature (34 °C) and then produced massively in a short term, but also the safety of it can be ensured effectively because of the clearly genetic background [11, 34]. For instance, in response of emergencies during the outbreaks of pdm09H1N1 in 2009, China had manufactured more than two emergency vaccines, and it had played a very positive role in rapid suspending of the spread in China [35].

## Conclusions

According to this study, we here put forward that when applying QIVs for vaccination, the recommendations of composition for influenza B could be altered and assessed once in 3 or 4 years. When economic burden was considered intensively and TIVs were utilized, the recommended compositions of the vaccines could be announced in April or May, rather than in February or March as now.

## Data Availability

Not applicable.
